# Factors Associated with Physical Fitness among Overweight and Non-Overweight Austrian Secondary School Students

**DOI:** 10.3390/ijerph16214117

**Published:** 2019-10-25

**Authors:** Gerhard Ruedl, Nikolaus Greier, Martin Niedermeier, Markus Posch, Vera Prünster, Martin Faulhaber, Martin Burtscher

**Affiliations:** 1 Department of Sport Science, University of Innsbruck, 6020 Innsbruck, Austria; nikolaus.greier@kph-es.at (N.G.); martin.niedermeier@uibk.ac.at (M.N.); markus.posch@uibk.ac.at (M.P.); vera.pruenster@student.uibk.ac.at (V.P.); martin.faulhaber@uibk.ac.at (M.F.); martin.burtscher@uibk.ac.at (M.B.); 2 University College of Education (KPH) Stams, 6422 Stams, Austria

**Keywords:** physical fitness, motor competence, physical activity, adolescents, overweight

## Abstract

The aim of the study was to evaluate factors associated with physical fitness (PF) among overweight and non-overweight Austrian secondary school students. PF was measured using the German motor test (DMT) 6–18 and students were asked about sex, migration background, weekly leisure time physical activity and daily electronic media use. In total 560 adolescents (56.6% female) with a mean age of 15.4 ± 1.4 years and a mean body mass index (BMI) of 22.5 ± 4.2 kg/m² were evaluated with 130 (23.2%) students classified as overweight or obese. For the total cohort, results of the multiple linear regression analysis (R^2^ = 0.34) revealed that a younger age (β = −0.16), no migration background (β = 0.13), a lower BMI (β = −0.29), a higher weekly leisure time physical activity (β = 0.34) and a lower daily electronic media use (β = −0.15) were associated with increased PF. Separate regression analyses for non-overweight and overweight students showed similar regression coefficients except for BMI. In conclusion, the positive influence of modifiable factors such as lower BMI, higher self-reported weekly leisure time sports activity and lower self-reported daily electronic media use should be considered already at young ages to increase physical fitness in overweight and non-overweight youth.

## 1. Introduction

Physical fitness, i.e., cardiovascular (cardiorespiratory, aerobic) fitness and muscular fitness, is an integrated measure of most, if not all, body functions involved in the performance of daily physical activity and/or physical exercise [1] and, therefore, also an important determinant of success for many popular sports and athletic events in youth [2]. In addition, physical fitness provides an important summative indicator of health, as there are strong associations with mortality and cancer in adults, independent of obesity and physical activity levels, as well as favorable associations with cardiometabolic disease risk, adiposity, mental health, cognition and bone health in children and adolescents [2,3]. Low cardiovascular and muscular fitness in adolescence are significantly associated with all-cause and cancer-associated mortality later in life [4,5]. Moreover, physical fitness is also related to brain structure and cognitive functions. Ruotsalainen et al. [6] recently found that aerobic fitness, but not (objectively measured) physical activity, is associated with grey matter volume. In addition, Marques et al. [7] reported in their systematic review that cardiorespiratory fitness was consistently and positively associated with academic achievement in children and adolescents aged 6–18 years, while objectively measured physical activity was inconsistently related to academic achievement.

There is some evidence that physical fitness in children and adolescents is inversely associated with current and future overweight and obesity [8]. This seems to be of utmost importance, because the prevalence of overweight and obesity among children and adolescents has dramatically increased in past decades in both developed as well as in developing countries [9]. Overweight and obesity at young ages are associated with increasing prevalence of cardiovascular risk factors, orthopedic problems and psychosocial constraints later on [10]. In addition, overweight and obesity lead to a reduced quality of life [11] as well as to a reduced life expectancy of overweight people by several years [12] and put a significant burden on the health care system [13].

Ortega et al. [14] found in a prospective study of about 600 normal-weight children over a duration of six years that improvements in fitness from childhood to adolescence are related with a reduced risk of becoming overweight or obese in adolescence. Werneck et al. [15] recently reported convincing association between increases in body adiposity from childhood to adolescence and the reduction in physical fitness in a three-year longitudinal study including about 370 children. In addition, Greier and Drenowatz [16] showed in a four-year longitudinal study of more than 200 Austrian middle school students a bidirectional, inverse association between body weight and physical fitness. Increased body weight was related with a decreased development of physical fitness and poor fitness levels increased the risk for excess weight gain. On the other hand, increased fitness was shown to contribute to weight loss in children, who were initially classified as being overweight or obese [16].

Physical fitness is partly genetically determined, but can also be greatly influenced by environmental factors [1]. There are several studies showing that physical fitness of children and adolescents depends on so-called modifiable factors, e.g., body mass index (BMI) or weight status, electronic media use and sports activity and on so-called non-modifiable factors as e.g., age, sex, socio-economic status or migration background [17,18,19,20]. In a study by Klein et al. [18] among a cohort of about 1400 German children and adolescents aged seven to 16 years, a higher socio-economic status was associated with a higher physical fitness compared to those with a lower socio-economic status. Regarding migration background, which is defined as present if children or at least one of their parents were born in a foreign country, Ruedl et al. [19] reported significant lower physical fitness of primary school children with migration background, which was only partly explained by a higher mean BMI in those children. In addition, Kaiser-Jovy et al. [21] found among a cohort of 391 Austrian adolescents aged 10 to 14 years that a high BMI, heavy media use, as well as migration background correlate negatively with both sport activity and physical fitness.

While there are some recent studies dealing with physical fitness and overweight of Austrian primary school children (ages 6–10 years) and secondary school children from grade 5 to 8 (ages 11–14 years) [16,20,21,22], less information is available regarding secondary school students from grade 9 to 12 (ages 15–18 years).

Thus, the aim of this study was to evaluate factors associated with physical fitness among Austrian secondary school students with a special focus on overweight and non-overweight adolescents.

## 2. Materials and Methods

Using a cross-sectional design, students from grade 9 to 12 (aged between 15 and 18 years) of four secondary schools located in the western part of Austria were tested from March to April 2018. The study protocol was approved by the school authorities of the federal state of Tyrol and by the board of each participating school. Parents were informed and gave informed consent. The study was conducted according to the ethical standards of the Declaration of Helsinki and was approved by the Board for Ethical Issues of the University of Innsbruck.

In accordance with our previous studies on Austrian children and adolescents [16,19,20,21,22] students’ height and weight were measured in sport clothing and barefoot. Body height was measured by using the mobile stadiometer “Seca 217” (Seca, Hamburg, Germany) with an accuracy of 0.1 cm and body weight was measured with the calibrated scale “Grundig PS 2010” (Grundig AG, Neu-Isenburg, Germany) with an accuracy of 0.1 kg. Based on these data, the BMI (kg/m^2^) was calculated and then students were classified according to the BMI reference system by Kromeyer-Hauschild et al. [23] into one out of five weight groups (anorexic, underweight, normal, overweight and obese). This reference system provides percentile curves separately for females and males aged 0–18 years to account for growth patterns due to age- and sex-specific differences [23]. The weight groups are based on the respective age- and sex-specific percentiles (e.g., overweight is defined as 90th to 97th percentile, obese is defined as 97th percentile and above) [23]. The reference sample consisted of a cohort of more than 34,000 German children and adolescents [23]. In addition, we classified students according to their weight status into two groups: overweight (including overweight and obese students) and non-overweight (including anorexic, underweight and normal weight students).

Physical fitness was assessed using the German Motor Test 6–18 [24], a standardized test battery consisting of eight items testing different subdomains of physical fitness: 20-m sprint (sprint velocity), balancing backwards on three 3 m long beams with different widths (coordination in a task requiring precision), jumping sideward over a middle line for 15 s (coordination under time pressure), stand-and-reach (flexibility), push-ups in a period of 40 s (strength endurance), sit ups in a period of 40 s (strength endurance), standing long jump (power) and 6-min run (endurance).

Values of the eight test items were standardized using the means and standard deviations of the norming sample with analogous age and sex according to Bös [24]. The guidelines of the test manual include multiplying the standardized score with 10 and adding 100 to obtain a so-called Z-score, where 100 equals to average performance in the tests. Z-values above 100 mean over-average performance and Z-values below 100 under-average performance. A total Z-score as an indicator for the overall physical fitness level of the students was built according to Bös [24]. The normal distribution of the total Z-scores was tested with the one sample Kolmogorov–Smirnov goodness of fit test.

According to Bös [24], the inter-rater reliability (0.95) and test–retest reliability (0.82) of the test battery were good, and the battery was validated for assessing speed, coordination, flexibility, strength and endurance. Combining the results of several subtests of the test battery to a single composite score for physical fitness was shown to be a valid approach [25]. However, it should be noted that the two tests “stand and reach” and “balancing backwards” are regarded as problematic. Utesch et al. [26] recommend eliminating the test “stand and reach” before calculating the composite score based on theoretical considerations on the structural model of motor abilities (i.e., passive mobility assessed by the test “stand and reach” is not considered a motor ability). Furthermore, the test “balancing backwards” might have less sensitivity in children older than eight or nine years [25,26]. Tests were carried out by specially trained physical education students in the sports halls at the participating schools under the exact instruction of the published test manual by Bös [24].

Students were asked about the mean duration (hours per day) of electronic media use (smartphone, tablet, computer, TV, etc.) outside of school and mean duration (hours per week) of practicing sports in a sports club as well as mean duration (hours per week) of engaging in physical activity during leisure time beside sports club participation. Weekly hours of sport club participation and weekly hours of physical activity in leisure time were summed up to get a total amount of weekly leisure time sports activity. As an indicator for migration background, students were asked according to previous studies [16,19] whether the language spoken at home was German (no migration background) or another one (migration background).

According to the distribution of data, independent *t*-tests or Mann–Whitney-U-tests were calculated to evaluate differences between the overweight and non-overweight students regarding mean total Z-score and mean Z-scores of any single test item as well as BMI, mean weekly leisure time sports activity and mean daily electronic media use. We calculated Cohen’s *d* as an effect size using the mean difference as numerator and the pooled standard deviation as denominator [27]. Positive values indicated higher physical fitness in non-overweight students; negative values indicated higher physical fitness in overweight students. Differences within sex and migration background between these two groups were evaluated by Chi-square-tests.

A multiple linear regression analysis was used to estimate the association of age, sex (female vs. male), migration background (yes vs. no), BMI, weekly leisure time sports activity and daily electronic media use with the dependent variable physical fitness (total Z-score) of the cohort. In addition, according to our previous study [20], separate multiple linear regression analyses for the non-overweight as well as for the overweight students were conducted entering the above-mentioned variables.

All *p*-values were two-tailed and values less than 0.05 were considered to indicate statistical significance. Data are presented as means ± standard deviations and absolute (relative) frequencies, respectively.

## 3. Results

In total, 560 students (56.6% female) with a mean age of 15.4 ± 1.4 years and a mean BMI of 22.5 ± 4.2 kg/m² were tested. According to the used reference system [23], five (0.9%) students were anorexic, 21 (3.8%) students were underweight, 404 (72.1%) had normal weight, 72 (12.9%) were overweight and 58 (10.4%) students were obese, respectively. With regard to the additional classification into only two weight groups, 76.8% of students were in the non-overweight group and 23.2% in the overweight group, respectively. A total of 151 (27.0%) students reported to have a migration background. Mean physical fitness according to total Z-score of all students was 105.2 ± 6.7. Mean weekly leisure time sports activity was 5.7 ± 4.9 h and mean daily electronic media use 2.8 ± 1.9 h, respectively.

Non-overweight students showed significantly higher values compared to overweight students in physical fitness (total Z-score) and all sub-disciplines with the exception of the stand-and-reach test, where no significant difference was found (Table 1). In addition, non-overweight and overweight students significantly differed with regard to percentage of migration background (22.3 vs. 42.6%, *p* < 0.001) and daily electronic media use (2.8 ± 1.9 vs. 3.0 ± 1.7 h, *p* = 0.026), but not significantly regarding sex (57.9 vs. 52.3% female, *p* = 0.268) and mean weekly leisure time sports activity (5.8 ± 4.8 vs. 5.3 ± 5.1 h, *p* = 0.108).

According to the multiple linear regression analysis for the total cohort (Table 2), the factors age, migration background, BMI, weekly leisure time sports activity and daily electronic media use showed a significant association with physical fitness (total Z-score) among this cohort of secondary school students. Higher physical fitness was associated with lower age (β = −0.16), lower BMI (β = −0.29), higher self-reported weekly leisure time sports activity (β = 0.34), lower electronic media use (β = −0.15) and no migration background (β = 0.13). Sex did not show a significant association with physical fitness. The model explained 34% of the variance of total Z-score. The model for non-overweight students showed a lower age (β = −0.14), no migration background (β = 0.15), a higher mean weekly leisure time sports activity (β = 0.38) and a lower mean daily electronic media use (β = −0.16) to be significantly associated with a higher total Z-score. The model explained 25% of the variance of the total Z-score. According to the model for overweight students, a lower age (β = −0.27), a lower BMI (β = −0.49) and a higher mean weekly leisure time sports activity (β = 0.22) was significantly associated with a higher total Z-score. The model explained 55% of the variance of the total Z-score.

## 4. Discussion

The main findings of the present study demonstrate for the total cohort of secondary school students a significant association between a higher physical fitness with a younger age, no migration background, a lower BMI, a higher weekly leisure time physical activity and a lower daily electronic media use. This was also true for the non-overweight group with the exception of the BMI. For the overweight group, a higher physical fitness was significantly associated with lower age, a lower BMI and a higher mean weekly leisure time sports activity.

The inverse relationship between age and physical fitness seems surprising, since physical fitness increases with maturation [18]. Although some motor abilities plateau at the age of adolescence (e.g., coordination, agility), other abilities still show an increase (e.g., endurance, strength-based abilities) [18]. Therefore, a positive relationship between age and physical fitness would be expected. However, it has to be noted that the total Z-score was used to operationalize physical fitness. The calculation of the total Z-score takes into account the mean and standard deviation of the age- and sex-matched norming sample [24] and can therefore be seen as a standardization of the fitness on age and sex. Thereby, a Z-score of 100 of both a 15-year-old and an 18-year-old student represent different physical fitness levels even though both Z-scores mean average physical fitness level regarding age and sex. Consequently, the negative relationship between age and the total Z-score found in the present study indicates that the physical fitness of younger Austrian adolescents deviated *more* from the age- and sex-matched norming sample compared to older adolescents, where the physical fitness was similar to the norming sample. The calculation of the Z-score can also explain why sex did not emerge as a significant variable in the regression models as the norming sample is sex-matched. In addition, with increasing grades in Austrian secondary schools the number of weekly lessons in physical education decreases. This might be also a potential explanation for this result as a higher number of weekly lessons in physical education is positively associated with a higher physical fitness [20].

Migration background in this study was significantly associated with a lower physical fitness in all three regression models and with a higher proportion of overweight, which is well in accordance with other studies [19,28]. Beside the higher prevalence of overweight, the lower fitness level of adolescents with migration background may be partly caused by an unfavorable and insufficient level of activity in leisure time as results of the MoMo-Study in Germany showed significant effects of a migration background on the level of physical activity in the leisure time of children and adolescents [29].

Regarding the BMI, there is evidence that a decrease in physical fitness is associated with an increase in body mass index among children and adolescents [30]. For example, in a pooled analysis including more than 73,000 adolescents aged 13–15 years, Garcia-Hermoso et al. [30] revealed low cardiorespiratory fitness and musculoskeletal fitness levels in overweight and obese adolescents when compared with their normal weight peers. The observed differences within physical fitness between overweight and non-overweight adolescents in this study are in line with previous research [17,20,22] and might be partly caused by the fact that excessive body fat of overweight and obese children and adolescents is an extra load to be moved during weight-bearing tasks [31]. However, the BMI cannot distinguish between lean and fat mass, and therefore provides no information of body fat distribution [32]. In addition, beside sex, the BMI depends on pubertal stage and ethnicity [32]. Especially during puberty, male adolescents increase their body weight due to rapid changes in muscle development. A meta-analysis on the diagnostic performance of BMI to identify obesity in children and adolescents showed a sensitivity of 73%, suggesting over a quarter of children not labelled as obese by BMI might indeed have excess adiposity [33]. To attenuate this potential bias we combined overweight and obese participants in a single group of overweight adolescents in this study. However, the percentage of about 23% overweight adolescents in this study is high compared to the expected 10% of the 90th percentile [23]. Therefore, we cannot exclude a misclassification of adolescents to the overweight group (e.g., adolescents with a large amount of muscle mass). It might be critically stated that a classification system of the year 2001 might be inadequate for year 2019 [23]. On the other hand, the classification system used allowed the reflection of increased prevalence of overweight compared to the reference sample [23], which is well documented in other studies [9]. Another cause for the lower fitness level of overweight children and adolescents might be a lower self-esteem [34] and a lower motivation to participate in physical activity which is influenced by their perceived and actual physical competence as well as by their parents’ perceptions of their physical competence [35]. The observed gap in strength and endurance capacities of overweight compared to non-overweight adolescents may contribute to the increased risk for cardiovascular diseases and orthopedic problems later on [10]. Woll et al. [17] found that obese children and adolescents had upper body strength and power values that were comparable or lower than normal-weight children and adolescents three or more years younger than them.

Self-reported weekly leisure time sports activity as an indicator for physical activity was positively associated with physical fitness within in the total cohort as well as within the non-overweight and overweight groups. Well in accordance, Marques et al. [36] revealed in a cohort of about 2500 adolescents aged 10–18 years that objectively measured time conducting moderate-to-vigorous physical activity was associated with higher physical fitness independent of sedentary time. Poitras et al. [37] reported in their systematic review that objectively measured physical activity is favorably associated with physical, psychological/social and cognitive health indicators whereupon these relationships are more consistent and robust for higher (e.g., moderate-to-vigorous) versus lower (e.g., light-intensity) physical activity.

A higher self-reported daily electronic media use was significantly associated with a lower physical fitness for the total cohort and for the non-overweight adolescents. This is well in accordance with the findings by Greier et al. [38], who recently showed that among a cohort of about 3300 adolescents, aged between six and 14 years, more TV time (>2 h per day) was associated with a significantly lower physical fitness. In addition, Drenowatz and Greier [39] found in a four-year longitudinal study of more than 200 Austrian middle-school students that high media consumption was associated with lower physical fitness. These results might be partly explained by the observation that in past decades, adolescents were becoming increasingly more inactive due to a higher engagement in sedentary activities such as watching TV or playing computer or interacting with their smartphone [40]. Recently, Kaiser-Jovy et al. [21] found among a cohort of about 400 Austrian 10–14 years old secondary school students a mean time of self-reported media use (watching TV, surfing in the internet, use of smartphone, playing computer etc.) of 10.3 h on a weekday and 12 h on weekends. However, as Kaiser-Jovy et al. [21] did not find a significant influence of hours of media use on sport activity, they concluded that a heavy media use is part of a complex juvenile leisure behavior and therefore rather a “time killer” with regard to sport activity and physical fitness. Notably, in a large-scale study including more than 100,000 adolescents, leisure-time sedentary behavior is not only associated with increased odds of obesity [41], but also associated with increased odds for feeling lonely [42] as well as with increased odds for suicide attempt in adolescence [43].

The magnitude of most of the standardized regression coefficients (age, sex, migration background, leisure time physical activity and electronic media use) can be considered similar for the three regression models. Differences regarding the significance of the independent variables might be explained by the sample sizes (overweight students: *N* = 130, non-overweight students: *N* = 430). Probably the largest discrepancy between the regression models is the role of the BMI. For the total cohort and for overweight students, the BMI showed a negative association with physical fitness, which is well in accordance with previously published results [30]. In non-overweight students, a (non-significant) positive association with physical fitness was found indicating that students with lower BMI show lower physical fitness. Although less discussed in literature, physical fitness is lower in underweight adolescents compared to their normal weight peers [30]. This might help to explain the differences in the slope of the regression lines. Another possible explanation is a non-linear relationship between BMI and physical fitness (e.g., J-shape relationship) as suggested by García-Hermoso et al. [30]. This explanation is supported by the higher amount of variance explained in the regression model for overweight students compared to the model for the total cohort indicating a more homogenous subsample in the overweight group.

Physical fitness and physical activity play an important role in the prevention of overweight and obesity in childhood and adolescence and reduces the risk of becoming overweight or obese in adulthood [44]. Thus, a special focus in school should be set on overweight and obese children and adolescents as early as possible. In a study among primary school children, more than two weekly lessons of physical education were associated with a higher physical fitness, and this factor was more pronounced among overweight compared to non-overweight pupils [20]. In addition, there is evidence that daily lessons in physical education reduce adiposity and show a significantly lower rise in BMI during primary school as well as increase motor abilities and decrease daily sedentary activities [45]. Thus, quantity (in the sense of volume) and quality (in the sense of moderate-to-vigorous intensity) of physical education lessons in school seem to be important factors to improve physical fitness in young ages for a healthier life later on.

Some limitations have to be considered when interpreting the findings of the present study. Firstly, the cross-sectional design did not allow causal interpretation of the results. Future prospective studies might reveal the predictive potential of the independent variables. Secondly, the majority of the independent variables were based on self-report of the students without the use of a validated questionnaire. Although some questions might be considered as face-valid (e.g., “Is the language spoken at home German?”), the psychometric criteria of the questionnaire remain to be elucidated. Furthermore, objective measures for assessing physical activity (e.g., pedometer or accelerometer) and for electronic media use (e.g., application measuring active time using smartphone) might be considered in future studies. Thirdly, we tested linear relationships for factors associated with physical fitness. Given the results of the separate regression models for overweight and non-overweight students in terms of the BMI, a non-linear model might be more appropriate. Fourthly, although often used in large cohort studies, e.g., by Garcia-Hermoso et al. [30], BMI does not differentiate between fat and nonfat mass or changes in body composition [46]. Furthermore, BMI does not take into account biological maturity status, which may vary largely during the time of adolescence [23,47]. Thereby, the usage of the BMI may have resulted in a misclassification of the individual weight status.

## 5. Conclusions

Taken together, physical fitness in this study among secondary school students was associated with age and migration background, BMI, self-reported weekly leisure time sports activity and self-reported daily electronic media use. The potentially positive influence of modifiable factors such as lower BMI, higher self-reported weekly leisure time sports activity and lower self-reported daily electronic media use should be considered already at young ages to increase physical fitness. Since the tested adolescents in this study spent up to 12 years in school, school is—independent of non-modifiable factors as age, gender and migration background or other socio-economic factors—an important place to improve physical fitness and to promote physical activity in children and youth.

## Figures and Tables

**Table 1:** Mean total Z-scores (±SD) and mean Z-scores (±SD) of the eight test items according to non-overweight and overweight students.

Z-Scores	Non-Overweight (*N* = 430)	Overweight (*N* = 130)	*p*-Value	*d*
Total	106.2 ± 5.9	101.7 ± 7.8	**<0.001**	**0.70**
20-m sprint	107.5 ± 10.1	102.0 ± 13.4	**<0.001**	**0.50**
Balancing backwards	106.2 ± 8.1	100.7 ± 10.7	**<0.001**	**0.63**
Jumping sideward	117.7 ± 7.8	114.6 ± 8.4	**<0.001**	**0.39**
Stand-and-reach	104.3 ± 10.2	104.6 ± 10.0	0.892	−0.03
Push-ups	111.1 ± 9.3	107.7 ± 12.0	**0.005**	**0.34**
Sit ups	97.3 ± 10.6	93.3 ± 11.4	**<0.001**	**0.37**
Standing long jump	105.5 ± 10.6	99.0 ± 11.7	**<0.001**	**0.60**
6-min run	99.2 ± 8.7	90.8 ± 10.5	**<0.001**	**0.92**

*d*: effect size Cohen’s *d*. Bold values indicate significant differences.

**Table 2:** Results of the multiple linear regression analysis of factors associated with physical fitness (total Z-score) in the total cohort, in non-overweight students and in overweight students.

	Total Cohort (*N* = 560) ^a^				Non-Overweight Students (*N* = 430) ^b^				Overweight Students (*N* = 130) ^c^			
Factor	B	SE B	β	*p*-Value	B	SE B	β	*p*-Value	B	SE B	β	*p*-Value
Constant	122.87	3.21		**<0.001**	106.10	3.96		**<0.001**	144.87	6.26		**<0.001**
Age (years)	−0.76	0.16	**−0.16**	**<0.001**	−0.60	0.18	**−0.14**	**0.001**	−1.31	0.33	**−0.27**	**<0.001**
Sex: 0 = female, 1 = male	0.09	0.49	0.01	0.857	0.08	0.53	0.01	0.879	0.10	1.08	0.01	0.926
Migration background: 0 = yes, 1 = no	1.87	0.55	**0.13**	**0.001**	2.06	0.62	**0.15**	**0.001**	1.67	1.00	0.11	0.098
BMI (kg/m²)	−0.47	0.06	**−0.29**	**<0.001**	0.20	0.12	0.07	0.104	−0.93	0.12	**−0.49**	**<0.001**
Weekly leisure time sports activity (h)	0.46	0.05	**0.34**	**<0.001**	0.46	0.06	**0.38**	**<0.001**	0.34	0.10	**0.22**	**0.001**
Daily electronic media use (h)	−0.52	0.13	**−0.15**	**<0.001**	−0.49	0.14	**−0.16**	**<0.001**	−0.47	0.31	−0.10	0.130

^a^ R = 0.582, R² = 0.338; ^b^ R = 0.499, R^2^ = 0.249; ^c^ R = 0.740, R^2^ = 0.548. B: unstandardized regression coefficient; SE B: standard error of unstandardized regression coefficient; β: standardized regression coefficient; BMI: body mass index; bold values indicate significant factors.
